# Preparation of Colon-Targeted Acetylharpagide Tablets and its Release Properties *in vivo* and *in vitro*

**DOI:** 10.3389/fphar.2018.00832

**Published:** 2018-08-14

**Authors:** DeWen Liu, Huijie Yan, Yiming Kong, Yun You, Yanling Li, Lixin Wang, Yan Tong, Jinyu Wang

**Affiliations:** Institute of Chinese Materia Medica, China Academy of Chinese Medical Sciences, Beijing, China

**Keywords:** colon-targeted tablet, acetylharpagide, release property *in vitro*, pharmacokinetic characteristics *in vivo*, *in vitro-in vivo* correlation

## Abstract

**Ethno Pharmacological Relevance:** Acetylharpagide is a monomeric compound extracted from *Ajuga decumbens*, widely used for remedying infectious and inflammatory diseases in Southern China.

**Aim of the Study:** The present study designed and investigated the formulation of colon-targeted acetylharpagide tablets according to the dual controlled release mechanisms of time-delay and pH-sensitivity.

**Materials and Methods:** The core tablets of acetylharpagide were coated with the material used in time-delay systems such as ethyl cellulose and suitable channeling agent, followed by pH-dependent polymers, polyacrylic resin II and III in a combination of 1:4. Furthermore, the release and absorption performance of colon-targets tables were evaluated *in vitro* and *in vivo*. In the *in vitro* tests, the optimized formulation was not released in simulated gastric fluid in 2 h; the release was <5% at pH 6.8 simulated intestinal fluids for 4 h; the drug was completely released within 5 h at pH 7.6 simulated colon fluid. In the *in vivo* tests, pharmacokinetic characteristics of the colon-targeted tablets were investigated in dogs.

**Results:** The results indicated that the acetylharpagide tablets with the technology of colon-targeting caused delayed T_max_, prolonged absorption time, lower C_max_, and AUCINF_obs. Meanwhile, the apparent volume of distribution (Vz_F_bs) of the colon-target tablets was higher than the reference.

**Conclusions:** These results suggested that colon-targeted acetylharpagide tablets deliver the drug to the colon. The *in vitro* performance of colon-targeted acetylharpagide tablet was appropriately correlated with its performance *in vivo*.

## Introduction

Ulcerative Colitis (UC) is an idiopathic inflammatory bowel disease, commonly occurring in developed countries in individuals aged between 15 and 30 years with an additional peak in the sixth decade of life. Despite improved understanding of the mechanisms of intestinal inflammation, the etiology, and pathogenesis of inflammatory bowel disease remain obscure. However, the heterogeneous disorders of multifactorial etiology in which hereditary (genetic) and environmental (microbial, behavioral) factors interact to produce the disease (Karlinger et al., 2000). Controlling inflammation and thus the symptoms, are the primary goals of treatment. However, the current therapies are only moderately effective, as several population-based studies have demonstrated that patients with UC have a 9–24% 10-year colectomy rate (Langholz et al., 1994; Nguyen et al., 2006; Jess et al., 2007), and a 33–45% twenty-five-year cumulative colectomy rate (Leijonmarck et al., 1990; Hoie et al., 2007). The symptoms of UC include rectal pain, rectal bleeding, and diarrhea; the treatment has to comprise of medications applicable to the rectum. This may include an enema, suppository, or foam. Rectal medications include 5-ASA (aminosalicylic acid), sulfasalazine or glucocorticoids (also known as steroids), which reduce the inflammation in the rectum and colon. About 25–40% of ulcerative colitis patients must eventually have their colons removed because of massive bleeding, severe illness, colon rupture, or the risk of cancer. Sometimes, the doctor will recommend removing the colon in the case of failure of the medical treatment or if the side effects of corticosteroids or other drugs threaten the patient's health (Langholz et al., 1994). Recently, oral colon-specific drug delivery system (OCDDS) gained considerable interests for the delivery of medications to treat diseases associated with the large intestine. Colon drug-targeting is valuable for the topical treatment of colon diseases such as Crohn's disease, ulcerative colitis, and colorectal cancer (Tiwari et al., 2012). The systemic adverse effects attributed to the drug could be minimized by limiting the drug absorption into the systemic circulation (Philip and Philip, 2010). The primary approaches to obtain colon-specific delivery included prodrugs, polymeric prodrugs, pH-dependent systems, time-dependent systems, biodegradable systems, and microflora activated systems (Zhou et al., 2009). The usage of altered pH is analogous to the more common enteric coating and consists of employing a polymer with an appropriate pH solubility profile. The concept of using pH as a trigger to release a drug in the colon is based on the pH conditions that vary continuously down the gastrointestinal tract (GIT). Several marketed formulations for treating inflammatory bowel disease have been reported, e.g., Prednisolone (Colal-Pred^®;^) is an oral colon-targeted pellet developed by Alizyme, Mesalazine(Asacol^®;^), Sulfasalazine (Azulfidine EN-tabs^®;^), and Prednisone (Rayos^®;^) are the delayed release tablets. Additionally, Mesalazine (Pentasa^®;^) are the timed release capsules (Amidon et al., 2015).

**Figure 1:**
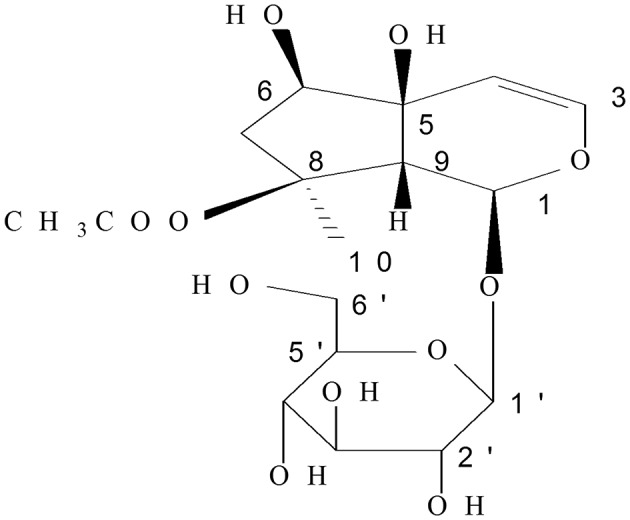
Chemical structure of acetylharpagide.

*Ajuga decumbens* Thunb. has been widely used as a remedy for infectious and inflammatory diseases in South China. Clinical observations indicated that *A. decumbens* could be utilized for cough phlegm (Jiangsu New Medical College, 1986; Konoshima et al., 2000), detumescence, detoxification, treating hepatitis, swollen furuncle, and trauma (1998), especially, as a traditional medicine for enteritis and diarrhea in the Fujian province of China (Wu, 1997). It is also reported to cure intestinal adhesion (Zhang, 1988) and intestinal fistula (Dinglin, 1987). Acetylharpagide is a monomeric compound of iridoid glycosides extracted from *A. decumbens* (Figure 1).

The iridoid glycosides, including acetylharpagide are reported to have anti-inflammation activity (Villasenor, 2007; Háznagy-Radnai et al., 2012), producing a series of allelochemical effects putatively associated with DNA synthesis (Pungitore et al., 2004). As shown in our previous study, acetylharpagide inhibited vascular endothelial cells migration, as well as, suppressed leukocytes adhesion and transmigration to endothelial cells under controlled shear stress *in vitro* (You et al., 2014). It is also reported that acetylharpagide could alleviate hyperfunction and effusion of capillary permeability during early inflammation (Tian et al., 2013), display significant antiphlogistic effects when administered *in vivo* in the carrageenan-induced paw edema test after intraperitoneal and oral administration in rats (Háznagy-Radnai et al., 2012). Acetylharpagide is a reference substance to control the content determination of *A. decumbens* with HPLC in the Chinese Pharmacopoeia 2015. In most cases, the reference substance is also one of the active substances. As a white amorphous powder with high hygroscopicity, acetylharpagide has excellent solubility in water and alcohol, but can be destroyed by the stomach acid because of the structure of hemiacetal (Liu et al., 2011). Hence, the current study aimed to design a novel colon specific drug delivery system containing acetylharpagide tablets. The usage of pH alterations is analogous to the common enteric coating and consists of a polymer with an appropriate pH solubility profile. The concept of using pH as a trigger to release a drug right in the colon is based on the pH conditions that vary continuously along the GI tract (Anisimova et al., 2000). Previously, we successfully formulated the tablet of brevisapin for the colonic delivery with Eudragit S100 and found adequate release at the predetermined time points (Li et al., 2013). However, when the basic remedy changed to acetylharpagide, the formulation could not fulfill the requirements of colon target because of the different properties between brevisapin and acetylharpagide. Thus, in the present study, we have attempted to formulate colon-targeted tablets of acetylharpagide with the dual mechanism, pH-dependency, and time-delay release. The core tablets were coated with frequently-used material in time-delay system such as the ethyl cellulose and suitable channeling agent followed by coating with pH dependent polymers such as polyacrylic resin II and polyacrylic resin III in various combinations. Moreover, we provided a detailed evaluation of colon-specific drug delivery systems both *in vitro* and in the Beagle dogs. To the best of our knowledge, this is the first report on the colon-targeted delivery of acetylharpagide with optimized *in vivo* behavior.

## Materials and methods

### Materials

Acetylharpagide was purified through macroporous resin columns after water extraction. The method is described in the Chinese patents, numbered as 200510085017.2 and 201110022703.0. The purity of acetylharpagide was more than 98% by HPLC. Standards of acetylharpagide and cinnamic acid were obtained from National Institutes for Food and Drug Control. Microcrystalline cellulose (MCC, MW ≈ 30,000), sodium carboxymethyl starch (CMS-Na, MW ≈ 5 × 10^5^~1 × 10^6^), hydroxypropyl methylcellulose (HPMC, MW ≈ 86,000), polyethylene glycol 6000 (PEG-6000, MW ≈ 6,000), and magnesium stearate were pharmaceutical grade and obtained from Beijing Fenglijingqiu Commerce and Trade Co. Ltd. Talcum powder, polyacrylic resin II (dissolved at pH ≥ 6.0, MW ≈ 1.5 × 10^5^), and polyacrylic resin III (dissolved at pH ≥ 7.0, MW ≈ 1.5 × 10^5^) were pharmaceutical grade and obtained from Huzhou Zhanwang Pharmaceutical Co. Ltd. Ethyl cellulose (EC,MW ≈ 20,000) was pharmaceutical grade and obtained from Colorcon (China). Diethyl phthalate (DEP) and triethyl citrate (TEC) were analysis grade and procured from A Johnson Matthey Company. All other chemicals and solvents were of the chromatographic and pharmaceutical grade.

### Animals

#### Animal preparation and surgical procedure

The study was approved by Research Ethics Committee of the Institute of Chinese Material Medica, China Academy of Chinese Medical Science, Beijing, China. All animal studies were carried out in accordance with the guidelines and regulation for the care and use of laboratory animal of Center for Laboratory Animal Care, China Academy of Chinese Medical Sciences.

Six male beagle dogs, similar in age (1.5 years) and weight (10.0 ± 0.5 kg), were obtained from Beijing Shahetongli Laboratory Animals Centre with animal license number SCXK (Beijing) 2010-0004. They were housed in individual cages and administered a standard diet and water *ad libitum*. All the animals were clinically healthy as well as hematologically and biochemically normal throughout the experimental period. Food, but not water, was withheld for 24 h before and after drug administration.

### Methods

#### Preparation of core tablets

After preliminary experiments, the optimum formulation of core tablets was obtained. The core tablets were composed of acetylharpagide (25%, w/w), MCC (66%, w/w), CMS-Na (8%, w/w), and magnesium stearate (1%, w/w). Briefly, acetylharpagide (25 mg/tablet) with MCC, CMS-Na, and magnesium stearate was blended in a high-speed mixer/granule actor (SINA Pharmaceutical Equipment Co., Shanghai, China) for 10 min. Approximately, 400 g of this mixture was granulated with 100 ml alcohol and dried for 2 h in an oven at 60°C. The core tablets with an average weight of 100 mg were compressed using a 5-station rotary tablet press (Tianfan Machinery Factory, Shanghai, China) fitted with 6 mm diameter normal biconcave punches and die sets. A relatively constant hardness of the tablet was held around 6 kg for compression.

#### Evaluation of core tablets

The hardness of the tablet was determined by using a Dr. Schleuniger hardness tester (Switzerland), which is expressed in kg/cm^2^. The friability of the tablet was determined using Tianfa Friabilator (Tianjin, China), which is expressed in percentage. Twenty tablets were initially weighed (W initial) and transferred into the friabilator that was operated at 25 rpm/min for 100 revolutions. The tablets were weighed again (W final) and the percentage of friability was then calculated by using the following formula: (F = W initial–W final/W initial × 100). For assessing the weight variation, Ch P 2015 procedure for uniformity of weight was followed, 20 tablets were randomly selected for individual weight evaluation and collectively on an electronic scale (Sartorius, Germany). The average weight of 1 tablet was determined from the collective weight. Disintegration time test was carried out using a method according to Ch P; 6 tablets were randomly selected from each batch and placed in the hanging basket of the disintegrating apparatus containing 500 mL distilled water maintained at 37 ± 1°C. The medium was stirred at 150 rpm and the time taken for the tablets to disintegrate was recorded. Release studies were performed using Ch P (Dissolution Apparatus 2-paddle method) at a rotation speed of 100 r·min^−1^ at 37 ± 0.5°C. 900 mL simulated colon fluid (SCF, phosphate buffer saline at pH 7.8) was tested in a dissolution apparatus (Hanson Research, USA). Each of the 6 tablets was placed into the dissolution cup, and the program was run for 45 min, 5 mL liquid medium samples were collected at predetermined time points (consecutively adding 5 mL new medium maintained at the same temperature).

#### Preparation of time-delay/enteric coating fluid

Time-delay coating fluid consisting of 3% (w/w) HPMC (E30) and 2% (w/w) PEG-6000 in ethanol were mixed by a magnetic stirrer and subsequently stirred for 3 h at 60°C to form a smooth dispersion (A). Another contained 3% (w/w) EC in ethanol composed of DEP (0.6%, w/w) as plasticizer, and PEG 6000 (0.3%, w/w) as channeling agent (B). The 2 solutions were mixed such as ensuring various adequate proportions. The coating thickness was 5%. The enteric coating solution was ethanol dispersions of various proportions of polyacrylic resin II and polyacrylic resin III. The plasticizer TEC (0.84%, w/w) and talcum powder (1.67%, w/w) were added to the polymer dispersions after homogeneous mixing.

#### Coating tablets

Put the core tablets in a rotary-drum coating machine (Mingxiang testing instrument Co., Shanghai, China) and open the rotation of the coating pan. The time-delay coating fluids were sprayed onto the surface of the core tablets slowly until reached the coating level. Then, the tablets with the first layer were dried in a drying cabinet for 12 h. The enteric coating fluid was sprayed onto the first layer tablets with the same method in the same machine. For the coating process, the coating pan was adjusted to the rotation speed of 30 r·min^−1^ at an angle of 45°, inlet air temperature of 40°C, and atomizing pressure of 0.4 kg·cm^−2^. The coating solution was sprayed onto the tablets at a flow rate of 5.5 mL·min^−1^ (Li et al., 2013).

#### *In vitro* release studies

Release studies were performed using Ch P (Dissolution Apparatus 2-paddle method) at a rotation speed of 100 r·min^−1^ at 37 ± 0.5°C. Briefly, 900 mL dissolution medium was tested in a dissolution apparatus (Hanson Research); Each of the 6 tablets was placed into the dissolution cup, and the release characteristics were evaluated by 3 sequential dissolution media. The dissolution was first run for 2 h in a medium of simulated gastric fluid (SGF, 0.1 mol·L^−1^ hydrochloric acid solution, pH 1.2) for 2 h in order to mimic the average gastric emptying time (Obitte et al., 2010). At the end of the 2 h, the equipment was switched off the paddle and rinsed to remove the previous medium after carefully removing the tablets; the dissolution medium of SGF was disposed and adequately rinsed with purified water. Then, a second dissolution medium, simulated intestinal fluid (SIF, phosphate buffer solution of pH 6.8, was emptied into the 900 mL container and the temperature allowed to attain 37 ± 0.5°C. The tablets were reinstated into the cup, and the dissolution run as before, but for 4 h as the reported average intestinal transit time is 3–4 h (Obitte et al., 2010). At the end of 4h, the medium was again removed and replaced with a third medium of simulated colon fluid (SCF, phosphate buffer solution of pH 7.8) and the same process was repeated for 5 h. Five milliliters liquid medium samples were collected at predetermined time points (at the same time, adding 5 mL new media with the same temperature). Acetylharpagide is a highly soluble drug, the dissolubility in SIF is 1.20 g/ml and in SCF is 1.26 g/ml, so all the *vitro* release tests carried out in sink conditions. High-performance liquid chromatography with Waters e2695 (USA) was used to determine the contents of acetylharpagide by a Welchrom ODS C_18_ stainless steel column (4.6 × 250 mm, 5 μm). The mobile phase consisted of acetonitrile-water (12:88, v/v), at a flow rate of 1.0 mL·min^−1^, the detection wavelength 207 nm, and the column temperature 30°C. The cumulative release at different time points was calculated, and the tablets were evaluated for drug release properties *in vitro*. In addition, to understand the mechanism of drug release, several release models were tested, such as Higuchi, zero order, and first order.

#### Pharmacokinetic study

Six beagle dogs were randomly assigned to one of two crossover experiments with a 10 d washout period. Food, but not water, was withheld for 24 h before and after drug administration. Each dog was orally administered either 8 core tablets (reference formulation) or 8 colon-targeted tablets (test formulation), respectively. Blood samples (3 mL each) were collected from the reference (0, 0.5, 1, 1.5, 2, 3, 4, 5, 6, 8, 10, 12, 24, 36, 48 h) and the test at predetermined times (0, 1, 2, 3, 4, 5, 6, 7, 8, 9, 10, 11, 12, 24, 36, 48 h). Plasma was immediately obtained by centrifuging the blood samples at 5,000 r·min^−1^ for 10 min and stored at −20°C until analysis. A 240 μL plasma sample was mixed with 960 μL of 6 ng·mL^−1^ cinnamic acid (internal standard) in a centrifuge tube. The solution was agitated for 30 s and then centrifuged at 15,000 r·min^−1^ for 10 min. The 1,000 μl organic layer was transferred to a clean tube and evaporated to dryness under nitrogen at 40°C. The residue was dissolved in 100 μL mobile phase. Ten microliters of the solution was injected into the LC-MS/MS system.

#### LC-MS/MS condition

The quantitative determination of acetylharpagide in plasma samples was performed on an LC-MS/MS equipped with Agilent 1200-6410B (MassHunter workstation, Agilent Co., USA) on a Welch Ultimate XB-C18 column (2.1 × 50 mm, 3 μm). The mobile phase consisted of acetonitrile-water (5:95, v/v) at a flow rate of 0.3 mL·min^−1^ and wavelength 207 nm. The column temperature was maintained at 30°C. The mass spectrometer was operated under electrospray ionization (ESI) with an ion spray voltage of +4,000 V. The positive ion multiple reaction monitoring (MRM) mode analysis was performed using nitrogen as the collision gas. The nebulizer gas (nitrogen) flow rates were adjusted to 3.0 L·min^−1^. The pressure in the collision cell was set at 35 psi. The precursor/product ion pairs for acetylharpagide and cinnamic acid (internal standard substance) were m/z 429.2/369.2 and m/z 149.2/103.1. The mass spectrometer was adjusted for optimum sensitivity and mass accuracy by infusion of a fresh standard solution of acetylharpagide at 0.23 ng·mL^−1^. The limit of detection measures from the calibration curve was 0.72 ng·mL^−1^. The intra-day precision and accuracy of the assay was measured by analyzing 3 different concentrations of spiked dog plasma samples at a concentration 2, 50, and 1,000 ng·mL^−1^. The intra-day accuracy of the method for acetylharpagide was ranged from 92.18–94.57% and the precision (%CV) ranged from 0.56–2.02%. The inter-day accuracy and precision were determined over 3 days by analyzing 3 spiked dog plasma samples at 2, 50, and 1,000 ng·mL^−1^. The precision expressed as the coefficient of variation (% CV) was 2.47, 1.67, and 2.11, respectively. The recovery of the acetylharpagide was evaluated by comparing the quantitative results of the spiked dog plasma samples with the aqueous standard solution of acetylharpagide in 3 different concentrations i.e., 2, 50, and 1,000 ng·mL^−1^ (*n* = 6 per concentration) in both media. The mean recovery was 85.4, 89.2, and 88.7%, respectively. The precision and accuracy of the method were both consistent with the analysis requirement. The specificity of the assay was demonstrated by obtaining ion chromatograms for blank dog plasma samples and blank dog plasma spiked with only the internal standard; no interference was detected from endogenous substances within the analytes and IS. The linearity of the method was studied over the concentration range of 1–5,000 ng·mL^−1^ in spiked dog plasma in triplicate by employing the standard calibration curves at least seven points. The method exhibits the linear response in the above range with correlation coefficient, *R*^2^ = 0.9994 and the regression equation, *Y* = 0.0021*X* + 0.3161. The stability of acetylharpagide in dog plasma was evaluated by analyzing the 3 different concentrations of spiked dog plasma samples at 2, 50, and 1,000 ng·mL^−1^, assessed over 12 h at room temperature by repeated freezing and thawing stored at room temperature and −20°C. The samples were then stored at −20°C for 6 months at 3 concentrations in duplicate in comparison with day 0. The precision expressed as the coefficient of variation (% CV) was 2.48–7.31, 5.63–10.47, and 8.95–13.28%, respectively.

#### Data analysis

The pharmacokinetic parameters were obtained using WinNonlin^®;^ (Version 6.3, Pharsight Corporation, USA) standard non-compartmental program. The *in vivo* absorption data of the drug for the colon-targeted tablet was calculated according to the Wagner-Nelson method.

(1) $$\begin{array}{l} {\text{F}}_{\text{a}}=\frac{{\text{C}}_{\text{t}}+kAUC_{0\;\sim \;t}}{kAUC_{0\;\sim \;\infty }}\times 100\% \end{array}$$

Correlation between *in vivo* drug absorption and *in vitro* release was then determined by plotting the mean percentage absorbed *in vivo* at 0.5, 1.0, 1.5, 2, 3, 4, 5, 6, 7, 8, 9, 10, 11, 12, and 24 h vs. the mean percentage released *in vitro* at the same time points. The similarity factor (*f* 2) adopted by the US Food and Drug Administration was used to evaluate the similarity in release profiles between the two pharmaceutical preparations (Ocaña et al., 2009). The similarity factor as a logarithmic transformation of the sum-squared error of differences between the absorbed *in vivo* and release *in vitro* was calculated by the following Equation (2):

(2) $$\begin{array}{l} f_{2}=50\times \log\left\{{\left[1+\frac{1}{n}\sum_{i=1}^{n}{\left({\text{vivo}}_{t}-{\text{vitro}}_{t}\right)}^{2}\right]}^{-0.5}\times 100\right\} \end{array}$$

Where vivo_*t*_ is the absorption date in beagle dogs, vitro_*t*_ is the accumulated release rate data in the dissolution medium at the predetermined time points respectively; n is the number of the time points. The similarity factor fits the result between 0 and 100. If *f*_2_ >50, the release profiles are considered to be similar. The larger the *f*_2_-value, the higher the similarity.

## Results and discussion

**Figure 2:**
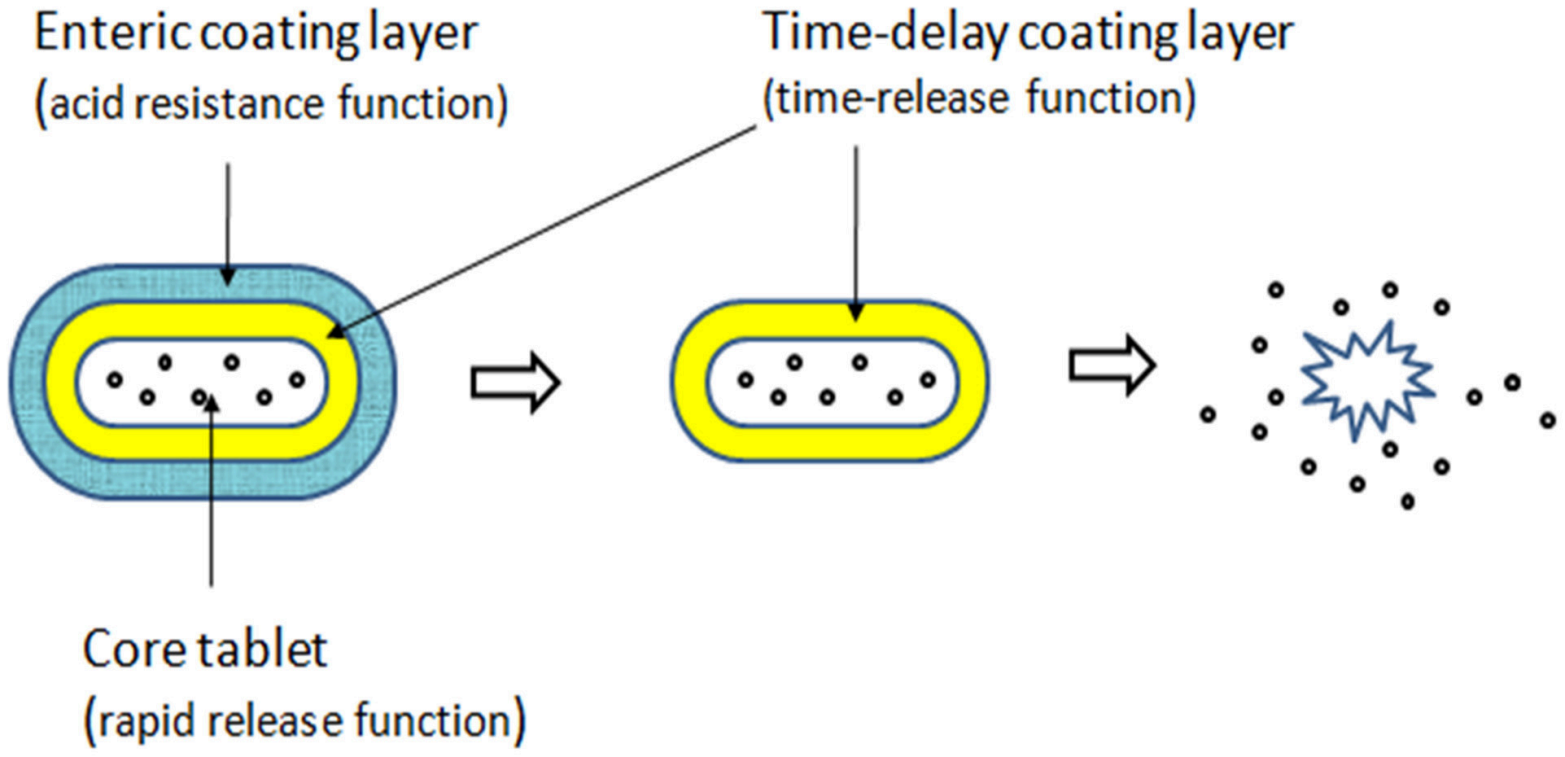
Tablets' structure diagram.

In our preliminary study, we designed pH sensitive colon-targeted tablets of acetylharpagide with various proportions of Eudragit^®;^ L100 and S100, which have been conventionally employed for coating the tablet and other formulations intended for colonic delivery (Khan et al., 2000; Asghar and Chandran, 2008a; Bhatt et al., 2014). We have also successfully formulated the tablet of brevisapin for the colonic delivery in a previous study (Li et al., 2013). However, that experiment failed when the drug turned into acetylharpagide; the tablets imbibed some dissolution medium, expanded, and formed small balls in 2 h followed by disintegration in simulated intestinal fluid (phosphate buffer solution of pH 6.8) at 3 h. The two potential reasons were as follows. First, acetylharpagide is highly soluble in water and hygroscopic; even in the slightest contact of water, the core tablet will imbibe the liquid and disintegrate. The second reason is that the polyacrylic resin could protect the drug from absorption in the environment of the upper GIT; however, the water vapor permeability test revealed the permeable coefficient for the film of polyacrylic resin as 0.056 (Mu et al., 1994). Thus, trace water will permeate the membrane through some tiny pores in it. Therefore, in the present study, we designed the dual layer coating tablets (Figure 2). The combination of pH-dependent polymers with time-base polymers could offer a means of achieving controlled release of the drug from the coated system (Akhgari et al., 2006). Application of polyacrylic resin in combination with ethylcellulose as multiunit layer tablets is an efficient method to achieve colon-specific delivery. Ethyl cellulose (EC) is an inert, hydrophobic polymer and has been extensively used as a retardant polymer for controlled release of a variety of drugs (Asghar and Chandran, 2008b). We chose EC as the inner layer to isolate the trace amount of water through a small aperture.

### Physical properties of core tablets

**Figure 3:**
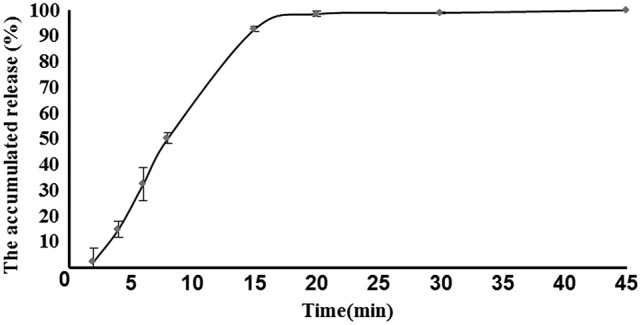
*In vitro* release of core tablets (*n* = 6).

The disintegration time, hardness, weight variation, and percentage friability of 3 batches of core tablets lied within the pharmacopeia limits and were given in Table 1. The *in vitro* release profiles of core tablets was depicted in Figure 3. All the results indicated that core tablets were satisfactory for appropriate matrix coating of acetylharpagide for colon-targeted delivery.

**Table 1:** Physical properties of core acetylharpagide tablets (*n* = 6).

**Batch**	**Disintegration time (min)**	**Hardness (N·cm^−2^)**	**Percentage friability (%)**	**Weight variation (%)**
1	5.3 ± 1.4	50.3 ± 0.8	0.11	1.17
2	4.7 ± 2.0	49.2 ± 1.2	0.13	1.22
3	4.2 ± 1.8	47.4 ± 1.6	0.08	0.53

### Proportion of time-delay coating fluid

The acetylharpagide core tablets were coated with the formulation consisting of part A and part B as mentioned in the methods section above. Other conditions were invariant. The *in vitro* release behaviors of the tablets with different ratio of A:B (1:6, 1:5, 1:4, and 1:3, w/w) were compared. The coating level exerted a significant impact on the acetylharpagide release from the coated tablets (Table 2). The four samples were rarely released in simulated gastric fluid after 2 h without distinct changes in their appearances. After 4 h release in simulated intestinal fluid, samples No. 3 and 4 solubilized more than 50%, while the coating layer of two samples broke. No. 1 and No. 2 were unbroken, and No. 2 solubilized 96.4% after 5 h in the simulated colon fluid.

**Table 2:** Effect of different proportion of time-delay coating fluid on acetylharpagide release from the coated tablets in three dissolution media (*n* = 6) (%).

**No**.	**A:B**	**Dissolve medium/Time**				
		**Simulated gastric fluid/**	**Simulated intestinal fluid/**	**Simulated colon fluid**		
		**2 h**	**4 h**	**1 h**	**4 h**	**5 h**
1	1: 6	0	0	0	32.1	54.6
2	1: 5	0	2.1	9.2	92.3	96.4
3	1: 4	0	58.7	89.6	94.4	94.5
4	1: 3	0	67.9	93.3	95.6	96.7

### Thickness of time-delay coating

Table 3 represents the release ratio of colon-targeting tablets with different level of time-delay coat thickness in three sequential dissolution media. The other conditions were similar with No. 2 in Table 2. The release of the drug was suppressed as it travels through the stomach, the data from No. 5 to No. 7 are all “0” in SGF. But the release of No. 5 was 46.2% in SIF, illustrating that the coated tablets have disintegrated in small intestine before they reached the colon. Compared No. 6 and No. 7, when the time-delay coating was 6%, the release ratio of acetylharpagide was 49.7% in simulated colon fluid, but when level was 5%, the release ratio was 96.2% in SCF. So, when the thick of coating is 5%, the release of the drug was suppressed as it travels through the stomach and the small intestine, and was allowed adequate entry into the colon. The results clearly demonstrated that the No. 6 was suitable.

**Table 3:** Effect of different thickness of time-delay coating on acetylharpagide release from the coated tablets in three dissolution media (*n* = 6) (%).

**No**.	**Coating level/%**	**Dissolve medium/Time**				
		**Simulated gastric fluid/**	**Simulated intestinal fluid/**	**Simulated colon fluid**		
		**2 h**	**4 h**	**1 h**	**4 h**	**5 h**
5	4	0	46.2	92.4	93.8	94.1
6	5	0	1.8	8.3	94.5	96.2
7	6	0	0	2.3	27.2	49.7

### Proportion of enteric coating fluid

The acetylharpagide release data (Table 4) showed the effect of the different proportion of polyacrylic resin II and III coating fluid. According to all the above experiments, sample No. 6 was selected as the optimal formulation, coated with a combination of polyacrylic resin II-polyacrylic resin III. The other conditions were invariable. Their release characteristics were examined by three sequential dissolution media. We investigated the effect of combination ratios on dissolution rate *in vitro*. As shown in Table 4, the combination ratio of polyacrylic resin II-polyacrylic resin III played a significant role in the release behavior of acetylharpagide colon-targeted tablets. Three samples were released 0% in simulated gastric fluid until 2 h. On the other hand, upon employing 1:3 (w/w) combination ratio of polyacrylic resin II-polyacrylic resin III as the coating formulation, the release ratio of acetylharpagide was 31.9% in simulated intestinal fluid. Thus the measurable release appeared. When the tablets were coated with polyacrylic resin II-polyacrylic resin III combination ratio of 1:4 (w/w), acetylharpagide was released 2.3% in simulated intestinal fluid at 4 h and 95.3% in simulated colon fluid at 5 h. When the ratio increased to 1:5, acetylharpagide was released 52.2% in simulated colon fluid at 5 h. Therefore, the sample No. 9 was selected as the best formulation.

**Table 4:** Effect of different proportion of polyacrylic resin II and III coating fluid on acetylharpagide release from the coated tablets in three dissolution media (*n* = 6) (%).

**No**.	**II:III**	**Dissolve medium/Time**				
		**Simulated gastric fluid/**	**Simulated intestinal fluid/**	**Simulated colon fluid**		
		**2 h**	**4 h**	**1 h**	**4 h**	**5 h**
8	1:3	0	31.9	66.5	95.1	95.1
9	1:4	0	2.3	15.6	95.2	95.3
10	1:5	0	0	4.4	36	52.2

### Thickness of enteric coating

When the combination ratio of polyacrylic resin II-polyacrylic resin III was maintained as 1:4 (w/w), the effects of the coating levels were examined from 5 to 10% on the release properties of acetylharpagide coated tablets. As shown in Table 5, when the coating level was adjusted as 5%, 12.1% of the drug was released in the simulated intestinal fluid that suggested the earlier measurable release. When the coating level increased to 10%, 90.3% of the drug was released until 5 h in simulated colon fluid. Consequently, the No. 12 formulation was the most appropriate thickness of the coating layer.

**Table 5:** Effect of different thickness of enteric coating on acetylharpagide release from the coated tablets in three dissolution media (*n* = 6) (%).

**No**.	**Coating level/%**	**Dissolve medium/Time**				
		**Simulated gastric fluid/**	**Simulated intestinal fluid/**	**Simulated colon fluid**		
		**2 h**	**4 h**	**1 h**	**4 h**	**5 h**
11	5	0	12.1	42.7	94.4	95
12	7	0	1.4	11.9	93.6	94.2
13	10	0	0	4.6	76.7	90.3

### *In vitro* release kinetics

As a successful colon-targeted tablet, the drug needs to be protected from absorption and/or the environment of the upper GIT and then be abruptly released into the proximal colon. In order to elucidate the underlying mechanism of drug release from colon-targeted tablet and core tablet, cumulative percentage released vs. time was plotted according to zero order kinetics, Higuchi model, and first order kinetics as illustrated in Figures 3, **5**. When the data were plotted according to the zero order equation, the core, and colon-targeted formulation showed a linearity with *R*^2^ 0.71 and 0.89; *R*^2^-value for Higuchi models was 0.83 and 0.91, respectively. When the same data were plotted according to the first order equation, complete linearity was observed with *R*^2^ 0.94 and 0.93. Therefore, from the present study, the preferred model was first order kinetics, which releases the drug such that it is proportional to the amount of drug remaining in its interior, similar to those containing water-soluble drugs in porous matrices (Costa and Sousa Lobo, 2001). It can be concluded that pH-based polymers in combination with EC can be used to form a dual layer tablet, which would prevent early drug loss from their matrices during upper GIT travel. It also confers that the drug could abruptly disintegrate and immediately act at the optimal site for colon-targeted delivery.

### *In vivo* absorption kinetics

**Figure 4:**
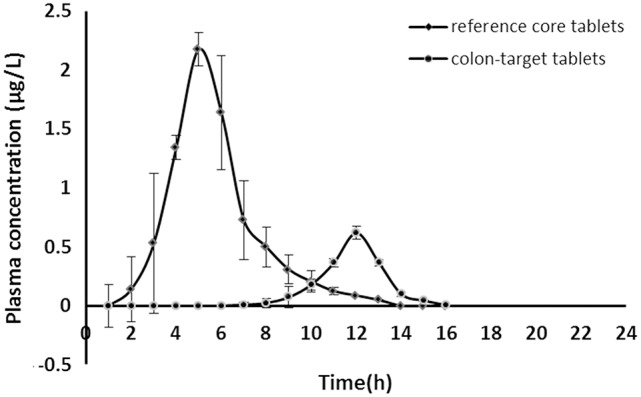
Acetylharpagide plasma concentration-time profiles after oral administration of colon-targeted tablets (●) and reference core tablets (♦) in dogs at a dose of 200 mg/body. Each value represents mean ± standard deviation (*n* = 6).

Either acetylharpagide test formulation (eight 25 mg colon-targeted tablets with coating) or acetylharpagide reference formulation (eight 25 mg core tablets without coating) was orally administered in the two crossovers experimental design in dogs at a dose of 200 mg/body, respectively. The concentration vs. time curve of the drug in plasma is illustrated in Figure 4.

The acetylharpagide appeared almost immediately within 0.5 h of oral administration of reference core tablets. The tablets rapidly disintegrated in the GIT and would have resulted in a quick absorption of the drug from the stomach and small intestine. Thus, a peak plasma concentration (C_max_) was achieved at 2.18 ± 0.10 μg·mL^−1^, T_max_ was 2 h as listed in Table 6. However, after oral administration of test colon-targeted tablets, acetylharpagide started appearing in the plasma at 6 h and reached C_max_ at 0.62 ± 0.03 μg·mL^−1^, T_max_ was 9 h. The results showed that the colon-targeted tablet did not release acetylharpagide in the stomach and small intestine. Upon reaching the colonic environment with pH 6.5–7.5, the acetylharpagide, colon-targeted tablets might disintegrate and release the drug contained in the formulation. Once the double coat layer dissolved, the core tablet of acetylharpagide might have disintegrated rapidly and produced a peak concentration of the drug by 9 h as shown in Figure 4. In Table 6, the pharmacokinetic parameters provided further evidence that the acetylharpagide colon-targeted tablet could be perfectly targeted to the colon. Absorption percentage *in vivo* of acetylharpagide for test formulation was assessed by non-compartment model. The mean residence time (MRT) of drug absorption from the test formulation was 9.34 h after oral administration, but the reference formulation was 3.18 h. The area under the plasma acetylharpagide concentration vs. time curves (AUC_INF_obs_) of the test and the reference formulations was 2.08 ± 0.22 μg·h^−1^·mL^−1^ and 6.37 ± 1.76 μg·h^−1^·mL^−1^, respectively. The relative bioavailability of the test formulation vs. reference formulation was 32.75%. The drug was absorbed at a relatively rapid rate in the stomach and small intestine (Krishnaiah et al., 2003). Because of the high water absorption capacity of the colon, the colonic contents are considerably viscous, and their mixing is not efficient. Thus the availability of most drugs to the absorptive membrane is low (Philip and Philip, 2010). The drug was absorbed slowly because of low permeability and less absorption surface area in the colon. Therefore, the acetylharpagide colon-targeted tablets release the drug resulted in lower C_max_ and AUC_INF_obs_ in the colon. Moreover, the apparent volume of distribution (Vz_F_obs) of the test was 42,891 ± 7,685 mL, and the reference was 7,151 ± 2,780 mL, respectively, which indicated that acetylharpagide was available for localized activity in the colon. The results of the pharmacokinetic evaluation in dogs showed that acetylharpagide colon-targeted tablets could target the drug to the colon. It was also evident from the *in vitro* drug release studies.

**Table 6:** Pharmacokinetic parameters after oral administration of acetylharpagide test colon-targeted tablets and reference core tablets in Beagle dogs.

**Parameter**	**Reference**	**Test**
Lambda_z (mL·h^−1^)	0.4963 ± 0.1439	0.2337 ± 0.0534
HL_Lambdaz (h)	1.49 ± 0.38	3.09 ± 0.66
T_max_ (h)	2.00 ± 0.00	9.00 ± 0.00
C_max_ (μg·mL^−1^)	2.18 ± 0.10	0.62 ± 0.03
AUC_last_ (μg·h·mL^−1^)	6.26 ± 1.73	2.05 ± 0.22
AUC_INF_obs_ (μg·h·mL^−1^)	6.37 ± 1.76	2.08 ± 0.22
Vz_F_obs (mL)	7151 ± 2780	42891 ± 7685
Cl_F_obs (mL·h^−1^)	3351 ± 928	9720 ± 1071
MRT_last_ (h)	3.18 ± 0.30	9.34 ± 0.41
MRT_INF_obs_ (h)	3.33 ± 0.39	9.57 ± 0.55

*Data are shown as mean ± standard deviation (n = 6)*.

### Correlation of *in vitro*/*in vivo* data

**Figure 5:**
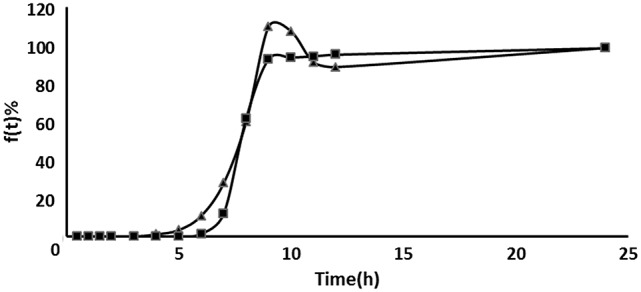
Comparison between the cumulative acetylharpagide release percentage *in vitro* (■) and the absorption percentage *in vivo* (▴) of colon-targeted tablets (*n* = 6).

**Figure 6:**
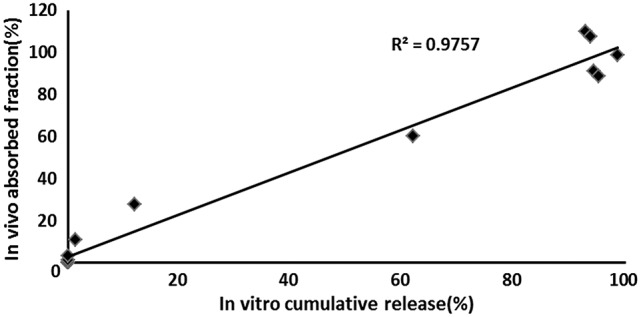
*In vivo*–*in vitro* correlation for colon-targeted tablets (*n* = 6). The symbol means the cumulative acetylharpagide release percentage *in vitro* (X axis) and the absorption percentage *in vivo* (Y axis) at the same time.

Both *in vivo* absorption data (F_a_) and *in vitro* cumulative release percentage of acetylharpagide vs. time profile demonstrated a similar contour curve with a significant correlation (*R*^2^ = 0.9757) for acetylharpagide colon-targeted tablet as illustrated in Figures 5, 6. The similarity factor between the absorbed *in vivo* and release *in vitro* was 51.1 calculated by the Equation (2). Therefore, it suggested an optimum association between *in vitro* release and *in vivo* absorption processes.

## Conclusions

In summary, the dual controlled release mechanisms of time-delay and pH sensitivity could successfully achieve colon-targeted drug delivery following oral administration. The equipment and technology are feasible for the manufacturing process. *In vitro* performance of colon-targeted acetylharpagide tablet was appropriately correlated with it's *in vivo* performance. An advantage of such a matrix design that comprises of pH-dependent polymers and hydrophobic polymer for colon-target delivery is that it can be applied to the hydrophilic compound, especially iridoid glycosides and phenolic acid, including other major medicinal drugs. However, the present study lacks the investigation on the gastrointestinal distribution of colon-targeted acetylharpagide tablets, for the beagle dogs that were not sacrificed. Furthermore, we aspire to investigate the tissue distribution in rats. In order to evaluate the applicability and clinical efficacy in the human body, further studies in healthy and patient volunteers are imperative.

## Ethics statement

All procedures involving animals in this study were performed according to the guidelines of the animal ethics regulations of The China Academy of Chinese Medical Sciences, Beijing, China.

## Author contributions

DL participated in literature search, study design, surgery operation, data collection, data analysis, data interpretation, and wrote the manuscript. HY and YK carried out the data collection and analysis, and provided the critical revision. YY, LW, and YT conceived of the study, and participated in its design and coordination. All authors read and approved the final manuscript. JW participated in study design and provided the critical revision. All authors read and approved the final manuscript.

### Conflict of interest statement

The authors declare that the research was conducted in the absence of any commercial or financial relationships that could be construed as a potential conflict of interest.
